# Pineal gland protects against chemically induced oral carcinogenesis and inhibits tumor progression in rats

**DOI:** 10.18632/oncotarget.27551

**Published:** 2020-05-19

**Authors:** Giseli Mitsuy Kayahara, Vitor Bonetti Valente, Rosani Belzunces Pereira, Felipe Yudi Kabeya Lopes, Marcelo Macedo Crivelini, Glauco Issamu Miyahara, Éder Ricardo Biasoli, Sandra Helena Penha Oliveira, Daniel Galera Bernabé

**Affiliations:** ^1^Psychoneuroimmunology Laboratory, Psychosomatic Research Center, Oral Oncology Center, São Paulo State University (Unesp), School of Dentistry, SP 15050-015, Araçatuba, São Paulo, Brazil; ^2^Department of Diagnosis and Surgery, São Paulo State University (Unesp), School of Dentistry, SP 15050-015, Araçatuba, São Paulo, Brazil; ^3^Laboratory of Immunopharmacology, Department of Basic Sciences, São Paulo State University (Unesp), School of Dentistry, SP 15050-015, Araçatuba, São Paulo, Brazil

**Keywords:** melatonin, cancer, oral cancer, carcinogenesis, pineal gland

## Abstract

Clinical investigations suggest that melatonin suppression and circadian dysfunction may be related to cancer development in shift workers. Studies also show that melatonin suppression after pinealectomy increases cancer incidence in preclinical models. However, no study evaluated the influence of pinealectomy on oral cancer development. In the current study, we investigated the effects of pinealectomy on oral squamous cell carcinoma (OSCC) occurrence and progression in rats. Rats submitted to sham surgery were used as control. Pinealectomy promoted an increase of 140% in OSCC occurrence when compared to sham animals. Tumors from pinealectomized rats displayed a higher volume and thickness than the tumors from sham-operated animals. Pinealectomy induced atrophy of the epithelium adjacent to the oral lesions. Pinealectomized rats showed higher mean number of tumor-associated macrophages and eosinophils in the invasive front of OSCC. In addition, nuclear overexpression of ERK1/2 and p53 was also observed in the front of carcinomas from pinealectomized rats. These results reveal that pineal gland plays a protective role against oral carcinogenesis. The melatonin suppression caused by the pinealectomy might contribute to oral cancer development by acting on ERK1/2 and p53 pathways and regulating tumor inflammation.

## INTRODUCTION

Currently, head and neck cancer (HNC) is the 6th most incident, while only oral cancer affects about 350.000 people worldwide [1]. Studies have shown that melatonin, a major hormone of pineal gland, may be used as adjuvant therapy for cancer treatment [2–4]. In the pineal gland, melatonin (N-acetyl-5-methoxytryptamine) is synthesized in the mitochondria from pinealocytes [5]. In response to darkness, its secretion is controlled by the suprachiasmatic nucleus, which relays photoperiodic information to the pineal via sympathetic nervous system [6]. In addition to the circadian rhythm control, pineal hormone has antioxidant, anti-inflammatory and oncostatic activities [7]. Melatonin may affect tumor growth by reducing cell proliferation and angiogenesis and inhibiting DNA damage [8]. A study showed that lung metastasis of gastric cancer may be inhibited by melatonin through the downregulation of MMP-2, MMP-9, and NF-κB [9]. Moreover, melatonin may increase the activity of tumor suppressor genes and modulate the proliferation and apoptosis of tumor cells through the p53 and ERK ½ signaling pathways [8, 10–12]. In Trp53−/− mice, melatonin administration inhibited lymphoma development [13]. Another recent study showed in an orthotopic model of oral cancer that melatonin administration reduced the p-ERK levels in the tumor microenvironment [14].

Inflammation plays a critical role in cancer onset and progression [15]. Liu et al. 2019 [16] demonstrated that cancer patients with high systemic inflammation index have lower disease-free survival and reduced distant metastasis-free survival when compared to patients with low inflammation levels. Systemic inflammation has also been associated with an increase in tumor invasion, greater risk of regional metastasis and advanced clinical stage in esophageal cancer patients [17]. Melatonin is considered an important molecule with anti-inflammatory features [18]. In ovarian tumors, the hormone may inhibit the release of proinflammatory cytokines TNF-α and IL6 in the tumor microenvironment [19]. Furthermore, melatonin can promote lower infiltration of eosinophils, IL-17+ inflammatory cells and Foxp3+ cells in the tumor tissue of hamsters with chemically induced cholangiocarcinoma [20].

Melatonin treatment may inhibit cancer progression and metastasis, as well as improve response of HNC patients to chemotherapy [2–4, 21]. Lissoni et al. [4] revealed that HNC patients treated with melatonin and chemotherapy displayed higher 1-year survival rate and increased tumor remission rate than those who received only chemotherapy. *In vitro* studies show that pineal hormone affects the motility of OSCC cell lines by inhibiting MMP-9 and VEGF transcription, which are molecules known to influence tumor progression [21, 22]. Although melatonin affects cancer progression, its role on the tumorigenesis is poorly known. Moreover, few investigations analyzed the effects of melatonin suppression after pinealectomy on cancer onset in preclinical models. In humans, melatonin suppression can occur in shift workers due to the artificial light exposure during the night periods. Pinealectomy avoids the release of melatonin from pineal gland and can be considered useful to assess the biological effects of pineal hormone suppression in preclinical models of diseases. Studies show that pinealectomized rats display higher incidence of DMBA-induced breast cancer than sham-operated animals [23, 24]. However, no studies have investigated the impact of pinealectomy on other types of cancer. In this research, we used a preclinical oral carcinogenesis model to test the hypothesis that the pinealectomy would promote higher chemically induced oral cancer occurrence and progression in rats.

## RESULTS

### Pinealectomy induces higher oral cancer occurrence and progression

**Figure 1:**
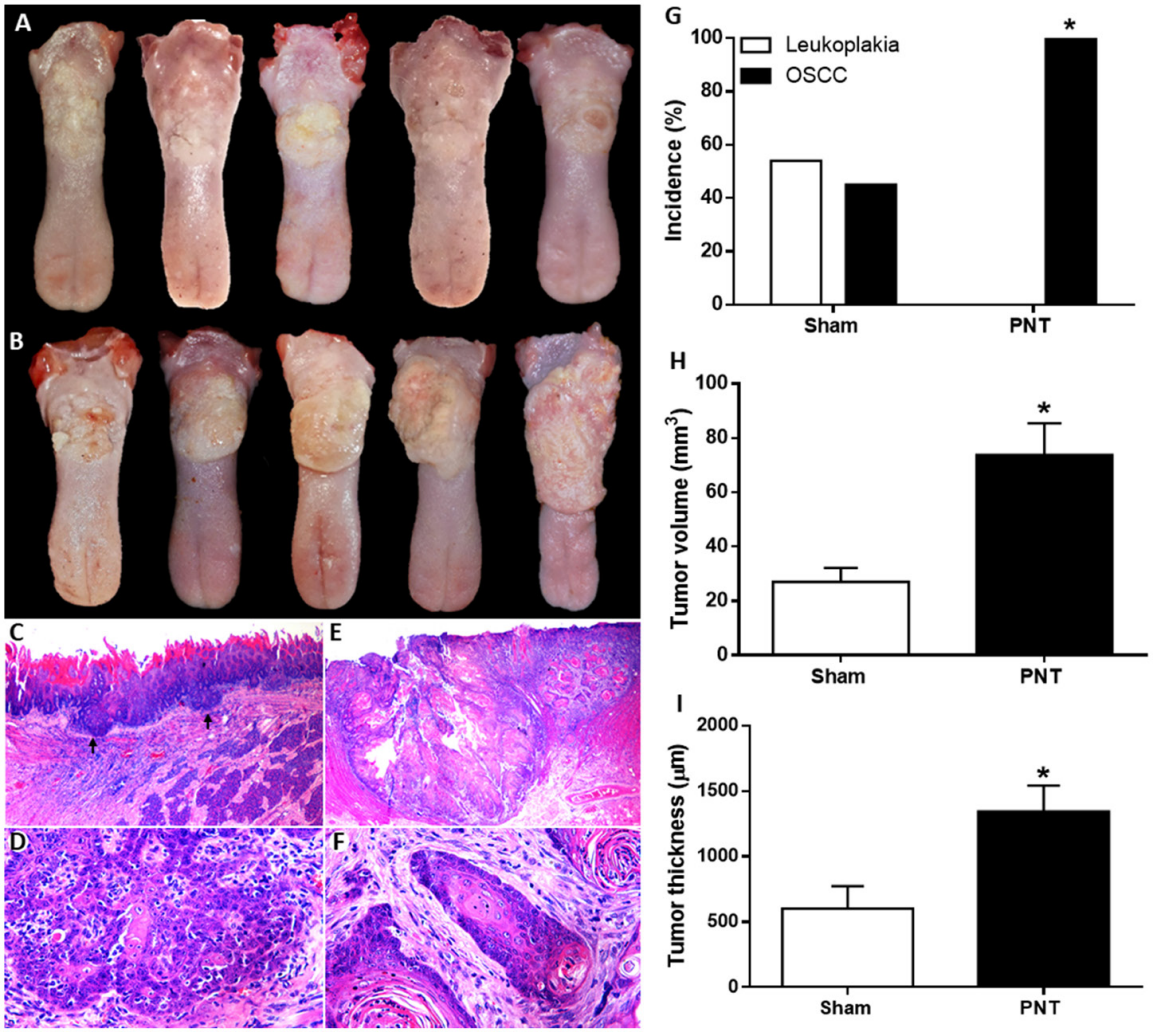
Clinical features of OSCCs derived from 4NQO treatment in sham (**A**) and PNT rats (**B**). (A) Small irregular white plates and discrete ulcerative lesions. (B) Extensive ulcerative lesions displaying reddish and yellowish-white areas. Histopathological features of tongue tumors from sham (**C** and **D**) and PNT rats (**E** and **F**) (H&E staining). (C) Well-differentiated OSCC (black arrows) (original magnification ×250). (D) Tumor cells showing hyperchromatism and dyskeratosis (original magnification ×400). (E) Extensive well-differentiated OSCC (original magnification ×250). (F) Islands of well-differentiated tumor cells with nuclear pleomorphism and keratin pearls (original magnification ×400). Occurrence and progression of OSCC in sham and PNT animals. (**G**) Chi-square test revealed that PNT rats had a higher OSCC occurrence than sham rats. (**H**) Student’s *t*-test showed that PNT group had increased tumor volume when compared to sham group. (**I**) PNT rats exhibited higher tumor thickness than sham-operated animals. (sham, *n* = 11; PNT, *n* = 12). Bars represent the mean ± SEM. ^*^ *p* < 0.05. The Figure 1E was spliced for joining together two images due to the extent of the tumor.

All pinealectomized (PNT) and sham rats were submitted to chemically induced carcinogenesis to investigate the effect of melatonin suppression on oral cancer occurrence. Clinical and histopathological features of OSCC from sham and PNT groups are showed in the Figure 1A–1F. After carcinogenic induction, PNT rats displayed higher OSCC occurrence than sham animals (*p* = 0.0029) (Figure 1G). According to World Health Organization (WHO) criteria, all PNT rats (100%) had well-differentiated OSCC. In the sham group, 54.5% displayed leukoplakia while only 45.4% developed well-differentiated OSCC. In addition, pinealectomy also promoted higher tumor growth. PNT rats exhibited higher OSCC volume and thickness than sham rats (*p* < 0.05). The tumor volume was about 3 times higher in PNT rats (73.76 ± 11.7 mm^3^) compared to sham animals (27.0 ± 5.19 mm^3^) (*p* = 0.0314) (Figure 1H). Furthermore, tumor thickness was twice greater in PNT rats (1344 ± 196.1 μm) than control animals (600.6 ± 172.7 μm) (*p* = 0.0341) (Figure 1I).

### Pinealectomy promotes atrophy of non-tumor oral epithelium

**Figure 2:**
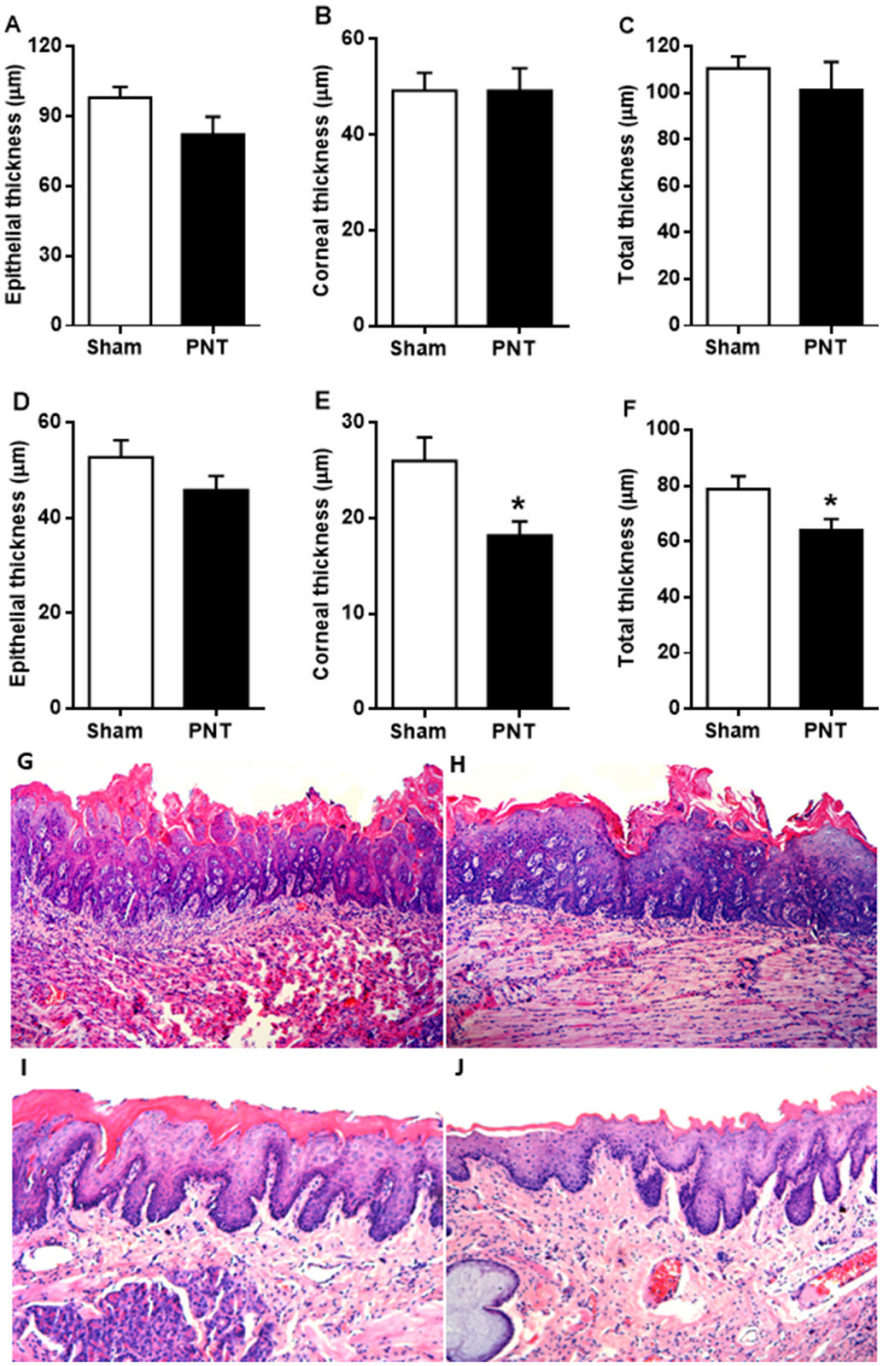
Non-tumor epithelial thickness adjacent to the tongue lesions derived from 4NQO treatment. (**A**–**C**) Student’s test showed no statistical differences between the groups regarding to epithelial, corneal and total thickness of non-tumor epithelium immediately adjacent to the tongue lesions. Epithelial thickness in distant sites from the lesion. (**D**) There were no differences between the groups in non-tumor oral epithelial thickness. (**E**) PNT rats displayed lower corneal thickness of non-tumor epithelium than sham-operated rats. (**F**) Student’s *t*-test revealed that rats from PNT group displayed reduced total thickness of non-tumor epithelium when compared to animals from sham group. (**G** and **H**) Total epithelial thickness immediately adjacent to the lesion from sham and PNT groups, respectively (H&E, original magnification ×100). (**I** and **J**) Total epithelial thickness distant from the lesion of sham and PNT animals, respectively (H&E, original magnification ×100). Bars represent the mean ± SEM. ^*^ *p* < 0.05 (sham-PNT, *n* = 10; PNT, *n* = 10). The background from histopathological photos were removed.

To assess the effects of pinealectomy on the morphologic features of non-tumor epithelium, the thickness of oral epithelium adjacent to the tongue lesions was measured (Figure 2G–2J). After carcinogenic induction, specimens from PNT and sham rats showed no differences regarding the epithelial thickness (PNT: 82.25 ± 7.628 μm *vs* sham: 98.10 ± 4.689 μm), keratin layer thickness (PNT: 49.23 ± 4.697 μm *vs* sham: 49.22 ± 3.739 μm) and total thickness (PNT: 101.3 ± 12.12 μm *vs* sham: 110.5 ± 5.195 μm) immediately adjacent to the oral lesion (*p* > 0.05) (Figure 2A–2C). Likewise, there was no statistical difference between the groups concerning the non-tumor oral epithelial thickness distant from the lesions (PNT: 45.81 ± 2.965 μm *vs* sham: 52.70 ± 3.596 μm) (*p* > 0.05) (Figure 2D). However, PNT rats displayed lower corneal thickness (18.19 ± 1.451 μm) than sham animals (25.98 ± 2.477 μm) (*p* = 0.0143) (Figure 2E). The total thickness of non-tumor oral epithelium also was reduced in the PNT rats (64 ± 4.007 μm) compared to sham animals (78.67 ± 4.725 μm) (*p* = 0.0292) (Figure 2F).

### Pinealectomy promotes increase of inflammatory cells in the invasive tumor front

**Figure 3:**
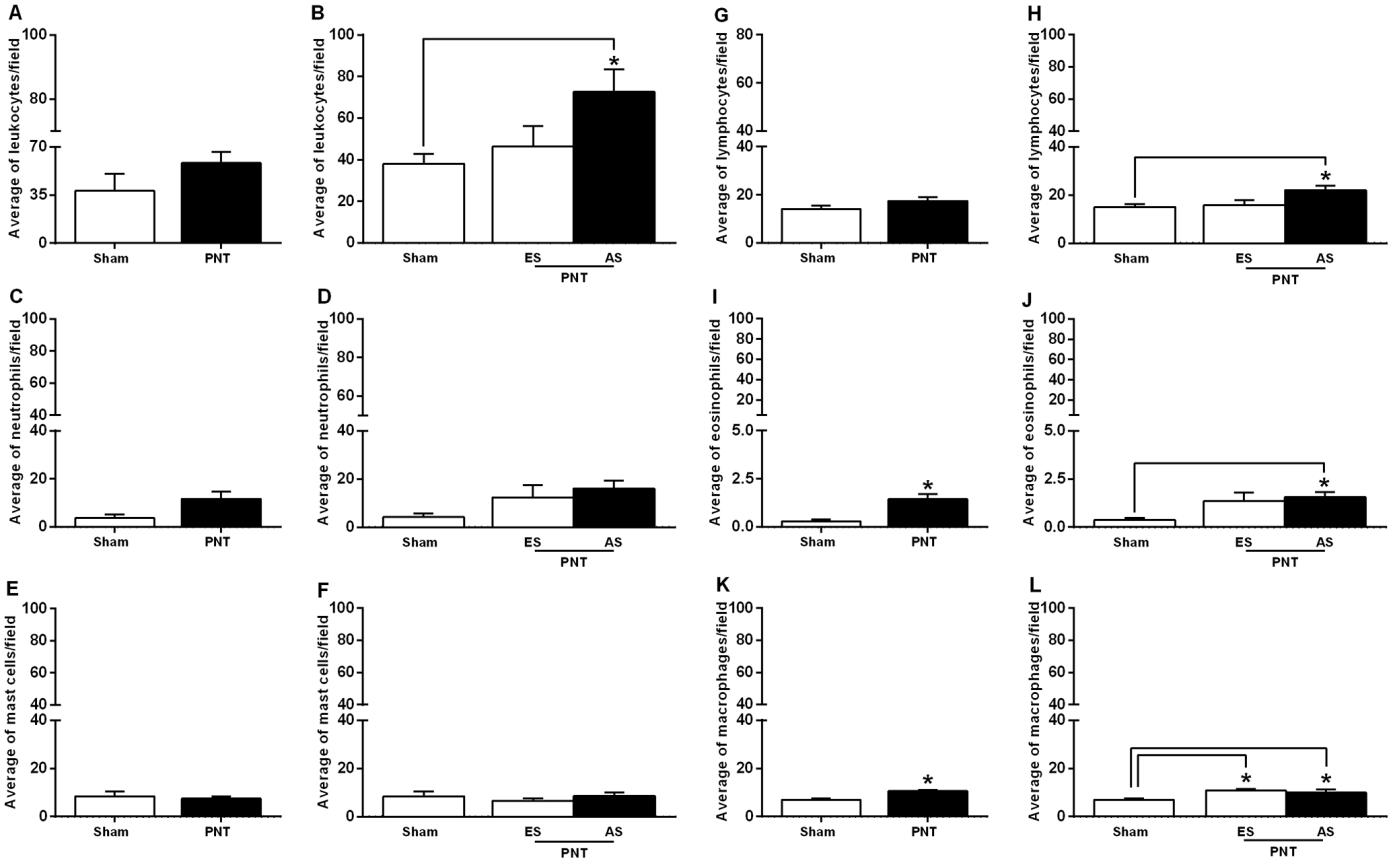
Inflammatory response in the tumor invasion front. Student’s *t*-test showed no differences in the average number of leukocytes (**A**), neutrophils (**C**), mast cells (**E**) and lymphocytes (**G**) in the OSCCs from sham and PNT animals. PNT rats displayed increased average number of tumor-associated eosinophils (**I**) and macrophages (**K**) compared to sham animals. In both groups, tumor size was not associated with the average number of neutrophils (**D**) and mast cells (**F**) in the tumor invasion front. Advanced tumor-bearing PNT rats had higher average number of leukocytes (**B**), lymphocytes (**H**) and eosinophils in the invasive front (**J**). Early and advanced tumor-bearing PNT rats exhibited increased average number of macrophages in the tumor invasion front than sham-operated rats (**L**). ^*^ *p* < 0.05. Bars represent the mean ± SEM. ^*^ *p* < 0.05. ES: Early stage. AS: Advanced stage. (sham, *n* = 5; PNT, *n* = 11).

The effects of pinealectomy on inflammatory response were evaluated in the tumor invasion front of the OSCCs from both groups. Student’s *t*-test showed that there was no significant difference between the groups concerning the average number of leukocytes (sham: 32.37 ± 4.104 cells/field *vs* PNT: 49.20 ± 6.368 cells/field) (*p* > 0.05) (Figure 3A). PNT rats displayed increased average number of eosinophils (PNT: 1.444 ± 0.2597 cells/field *vs* sham: 0.2800 ± 0.1020 cells/field) (*p* = 0.0074) (Figure 3I) and macrophages (PNT: 10.53 ± 0.6379 cells/field *vs* sham: 6.924 ± 0.5910 cells/field) (*p* = 0.0014) (Figure 3K) compared to sham animals. However, there were no significant differences between both groups regarding the average number of neutrophils (PNT: 11.74 ± 2.931 cells/field *vs* sham: 3.695 ± 1.434 cells/field) (*p* > 0.05) (Figure 3C), mast cells (PNT: 7.545 ± 0.8918 cells/field *vs* sham, 8.438 ± 2.014 cells/field) (*p* > 0.05) (Figure 3E) and lymphocytes (PNT: 17.45 ± 1.604 cells/field *vs* sham: 14.14 ± 1.358 cells/field) (*p* > 0.05) (Figure 3G). In our results, PNT rats displayed tumor volume approximately three times higher than sham group. However, some PNT animals had incipient lesions. Thus, to also examine the association between tumor stage and number of inflammatory cells in the invasive tumor front, carcinomas from PNT rats were classified into early or advanced stages. This classification was made based on a median split of the tumor volume. Advanced tumor-bearing PNT rats displayed higher average number of leukocytes (*p* = 0.0308) (Figure 3B), lymphocytes (*p* = 0.0341) (Figure 3H) and eosinophils (*p* = 0.0277) (Figure 3J) in the tumor invasion front than sham rats. There were no differences in the average number of neutrophils (Figure 3D) and mast cells (Figure 3F) concerning the tumor stage between PNT and sham rats (*p* > 0.05). Independently of tumor stage, mean number of macrophages was increased in the invasive tumor front from PNT rats (*p* = 0.0056) (Figure 3L).

### PNT rats display higher p53 and ERK1/2 expression in the tumor invasion front

**Figure 4:**
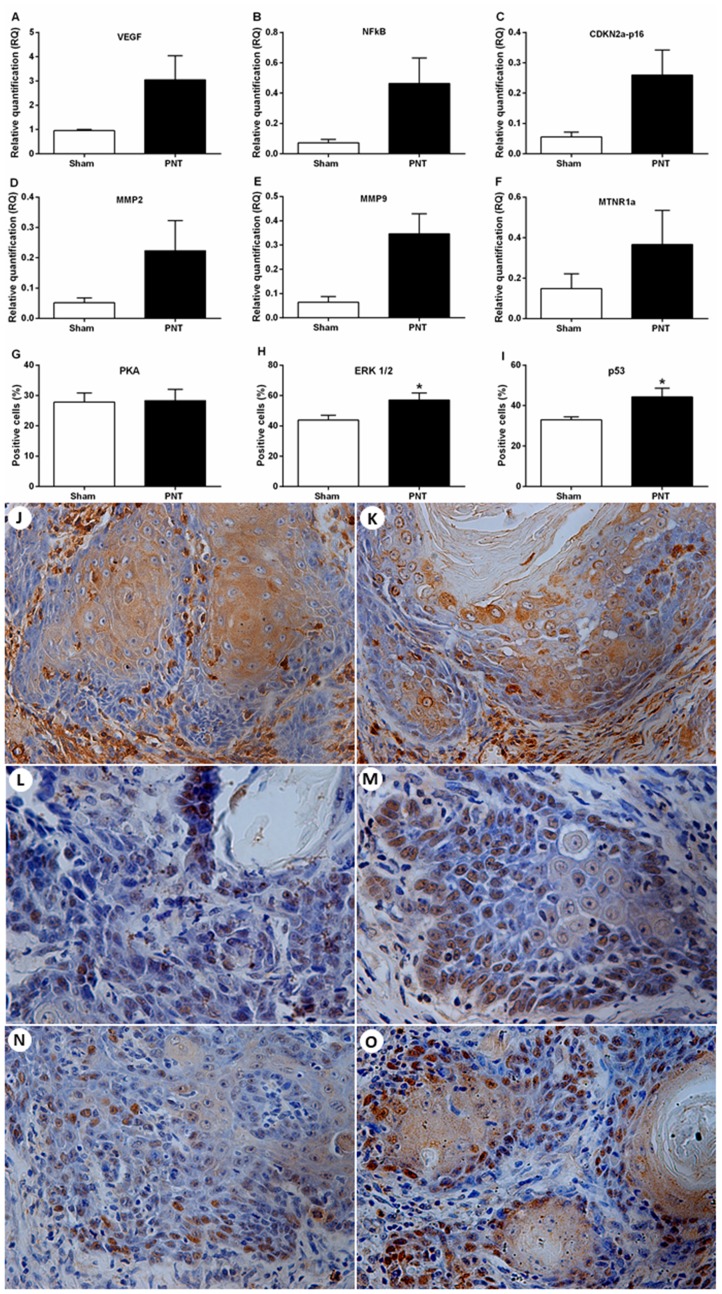
Expression of tumor progression-related genes and melatonin receptors and immunostaining for PKA, ERK1/2 and p53 in the OSCC microenvironment. (**A**–**F**) Student’s *t*-test showed no differences between the groups concerning the mRNA expression for VEGF, NFkB, CDKN2A-p16, MMP2, MMP9 and MTNR1a in the tumor microenvironment. (**G**) Student’s *t*-test showed no statistical differences between both groups for PKA expression in the OSCCs. (**H**) PNT rats had increased tumor expression of ERK1/2 compared to sham animals. (**I**) PNT rats displayed higher tumor expression of nuclear p53 than sham rats. Immunoexpression of PKA (**J** and **K**), ERK1/2 (**L** and **M**) and p53 (**N** and **O**) in the OSCC invasion front from sham and PNT rats, respectively (original magnification ×400). Bars represent the mean ± SEM. ^*^ *p* < 0.05. (sham-PNT, *n* = 7; PNT, *n* = 9).

The expression of tumor progression-related genes, melatonin receptors and carcinogenesis-related proteins were evaluated in the tumor microenvironment from sham and PNT rats. The mRNA levels of VEGF, NFkB, MMP2, MMP9, CDKN2a-p16 and MTNR1a were considerably higher in the carcinomas from PNT rats. However, these results did not reach statistical significance (*p* > 0.05) (Figure 4A–4F). MTNR1b was expressed only in three samples, two from sham group and one from PNT. In the invasive tumor front, there was no difference regarding the PKA immunoexpression between PNT (28.33 ± 3.709%) and sham (27.80 ± 3.001%) rats (*p* > 0.05) (Figure 4G, 4J, 4K). ERK1/2 immunoexpression was increased in tumor front from PNT animals (57,05 ± 4,645%), compared to sham rats (43,87 ± 3,177%) (*p* = 0.0451) (Figure 4H, 4L, 4M). Similarly, PNT rats exhibited higher p53 expression in the tumor invasion front (44,35 ± 4,330%) than sham rats (32,99 ± 1,438%) (*p* = 0.0430) (Figure 4I, 4N, 4O).

### Pinealectomy does not influence the depressive- and anxiety-like behaviors in rats that underwent oral carcinogenesis

**Figure 5:**
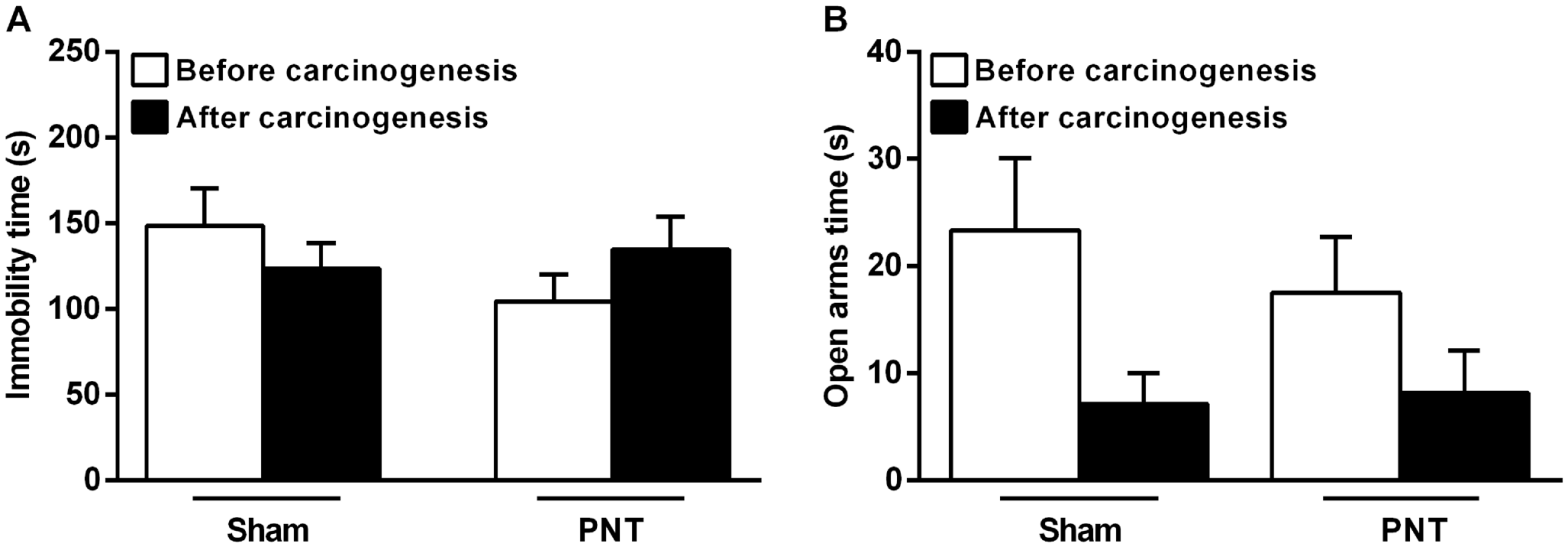
Depressive- and anxiety-like behaviors from sham and PNT rats. (**A**) There were no differences in the depressive-like behavior from sham and PNT rats pre- and post-carcinogenesis. (**B**) Student’s *t*-test revealed no differences between the groups regarding the anxiety-like behavior before and after carcinogenesis. Bars represent the mean ± SEM. *p* > 0.05. (sham, *n* = 11; PNT, *n* = 12).

To evaluate the behavioral alterations induced by the pinealectomy, all rats were tested for depressive- and anxiety-like behaviors through Forced Swimming Test (FST) and Elevated Zero Maze (EZM), respectively. Although PNT rats have displayed low-depressive (immobility time in FST, 104.3 ± 16.01s) and high-anxious behavior (time in the open arms in EZM test, 13.9 ± 4.105s) compared to sham animals (164.0 ± 28.08s and 21.0 ± 3.719s, respectively), both tests did not reveal statistical differences between the groups (*p* > 0.05) (Figure 5A and 5B). After carcinogenesis, there were also no differences between PNT and sham groups concerning the depressive- (PNT, 134.8 ± 19.11s *vs* sham,145.0 ± 11.29s) and anxiety-like (PNT, 12.08 ± 5.28s *vs* sham, 17.00 ± 7.49s) behavioral phenotype (*p* > 0.05) (Figure 5A and 5B).

## DISCUSSION

In order to evaluate the role of pineal gland and melatonin suppression on oral cancer occurrence and progression in rats, we used a classical pinealectomy model to induce the endogenous melatonin suppression. It is known that pinealectomy abolishes circadian production of pineal hormone and reduces the serum melatonin levels in different animal species [25–31]. Our results provided the first evidences that pinealectomy followed by melatonin suppression may influence oral cancer onset and progression.

In the current study, rats submitted to pinealectomy displayed increased OSCC incidence compared to rats that underwent sham-surgery. Moreover, PNT rats exhibited higher OSCC volume and thickness than sham animals. There are few evidences that show the impact of pinealectomy on cancer progression. Only El-Domeiri & Das Gupta [32] showed that pinealectomy resulted in higher melanoma growth in hamsters. Otherwise, a growing number of studies have investigated the effects of pineal gland hormone on cancer progression and treatment [4, 33]. Some studies show that melatonin has oncostatic effects such as the ability to inhibit angiogenesis, cell proliferation and metastatic processes [21, 22]. Melatonin metabolism may also affect cancer growth. Mitochondrial cytochrome P450 1B1 (CP4501B1) metabolizes melatonin to N-acetylserotonin (NAS) into tumor cells, which can induce apoptosis [5]. Furthermore, antitumor mechanisms of melatonin may modulate glucose metabolism [34]. Tumor cells, even in aerobic conditions, convert glucose to lactate (Warburg effect). Warburg effect enables the synthesis of macromolecules that are required for increased cellular proliferation, tumor growth and metastasis [34]. In this context, pineal gland hormone can inhibit the Warburg effect acting as a signal for cancer cells switch from glycolysis to mitochondrial oxidative phosphorylation [34, 35]. This mechanism has been explored in breast cancer cells and can contribute to reduce tumor progression [34]. Nevertheless, further studies are needed to assess whether the melatonin also inhibits Warburg effect in oral cancer cells.

Several other signaling pathways have also been investigated in relation to melatonin and OSCC. Liu et al. 2018 [14], for example, demonstrated that pineal hormone inhibits the OSCC growth by inactivating ROS-dependet Akt pathway and reducing the expression of cyclin D1, PCNA and Bcl-2. Both mechanisms result in lower cell proliferation and decreased epithelial-mesenchymal transition [14]. Pineal hormone also decreases the expression of pro-angiogenic genes HIF-1α and VEGF and ROCK-1 pro-metastatic gene [22]. In addition, it promotes transcriptional suppression of the MMP-9 gene, event mediated by reduced histone acetylation [21]. Thus, we suggest that pinealectomy induces opposite effects to the melatonin treatment in tumor cells promoting cell proliferation and increased OSCC progression. Studies have also shown that decreased levels of melatonin may be a risk factor for mammary cancer onset [23, 24, 36]. Nevertheless, to the best of our knowledge, this is the first study that demonstrates the influence of pinealectomy and pineal hormone suppression on oral carcinogenesis.

Pinealectomy can also promote morphometric and biochemical changes in the skin epithelial tissue, such as atrophy of the epithelium and decreased levels of antioxidant enzymes [37]. Here, we hypothesized that the molecular events related to oral carcinogenesis result from pinealectomy and can be accompanied by morphological changes in the oral epithelium. Our results showed that immediately adjacent to the tongue lesions, PNT rats displayed a lower epithelial thickness than sham animals, but this result was not significant. The analysis could have been influenced by the intense cell proliferation that normally occurs near the tumor. In PNT rats, distant regions from the lesion site, exhibited atrophy of corneal layer and total epithelial thickness. Pineal hormone is a powerful free radical scavenger [6]. Unlike, melatonin suppression promotes increase of reactive oxygen species (ROS) concentrations and reduces the levels of antioxidant enzymes in the skin of rats [37]. In non-transformed cells, high levels of ROS tend to promote cell growth arrest and/or cell death [38]. It suggests that the epithelial atrophy displayed by PNT rats may have be caused by a possible increase in the ROS levels into tongue microenvironment, which resulted in a lower proliferation of keratinocytes. The decreased epithelial thickness is associated to higher susceptibility of the mucosa to the penetration of carcinogenic substances [39]. Valentine et al. [40] showed that predisposing factors for oral cancer development such as increased alcohol and tobacco consumption were associated to reduction of the tongue epithelial thickness. Therefore, a lower epithelial thickness in PNT rats would allow a higher infiltration of 4NQO carcinogen through the tongue mucosa leading to increased DNA damage and malignant transformation of the keratinocytes.

Cancer development and progression may be influenced by inflammatory responses [15]. Immune cells can have dual effects on tumorigenesis, eliminating tumor cells or promoting cancer growth [41]. Our results demonstrated that the pineal gland hormone suppression increased the average number of eosinophils and macrophages in the tumor microenvironment. Moreover, large tumors from PNT rats had increased mean number of lymphocytes in the tumor invasion front. Likewise, previous investigations with non-cancer preclinical model showed that pinealectomy promoted an increase in the number of eosinophils and macrophages into peripheral tissues [39, 42]. In a cancer model, melatonin administration suppressed the mean number of eosinophils in cholangiocarcinoma samples [20]. The presence of eosinophils in the breast cancer microenvironment enhances tumor growth and lung metastasis [43]. In human OSCC specimens, tumor-associated macrophages have been positively correlated to tumor size, nodal metastasis, invasive behavior and invasive depth [44, 45]. In addition, tumor-associated eosinophilia has been associated with advanced disease stage and tumor invasion depth [44, 45]. In PNT rats, our findings strongly suggest that the pineal hormone suppression induced increase of inflammatory cells in the tumor microenvironment contributing to OSCC progression.

Cancer progression may be modulated by melatonin receptor-dependent mechanisms [46]. In the current study, mRNA expression for MTNR1a and MTNR1b genes was detected in the tumor microenvironment. MTNR1a and MTNR1b encode MT1 and MT2 receptors, respectively [47, 48]. MTNR1a and MTNR1b genes may be expressed in normal tongue microenvironment and OSCC cells [47, 48]. However, in oral cancer cells, melatonin receptors may suffer epigenetic silencing resulting in a lower tissue expression and increased tumor growth [48]. Silencing of MTNR1a gene in OSCC specimens has been correlated to tumor size and shorter overall survival [48]. The expression of MTNR1b in only some specimens of our samples may be associated to genetic alterations that would result in the gene silencing. Investigations have also shown that melatonin may inhibit the expression of tumor progression-related genes [49, 50]. A recent study demonstrated that NFkB expression, for example, can be reduced in breast cancer cells treated with melatonin [50]. In ovarian cancer patients, melatonin treatment may control cell invasion and metastasis by decrease in MMP9 activity [21, 22]. There are still no studies that have evaluated the expression of cancer progression-related genes and proteins in tumors from pinealectomized rats. In our study, mRNA levels for VEGF, NFkB, CDKN2a-p16, MMP2, MMP9 genes were increased in the tumor microenvironment from PNT rats, but the results were not significant because of the high cell heterogeneity found between OSCC samples of both groups. Higher OSCCs displayed a large quantity of tumor cells, whereas lower tumors also exhibited several stromal cells, such as inflammatory cells and fibroblasts, and blood vessels.

Several signaling pathways related to cancer progression may be modulated by melatonin. In our research, pineal hormone suppression did not change the PKA expression in OSCCs. On the other hand, our results showed that PNT rats display increased ERK1/2 expression in the tumor invasion front. Modulation of MAPKs pathways may be involved in anticancer properties of pineal hormone [11]. ERK1/2 belongs to a MAPK pathway and may control several cell responses, such as proliferation, migration, differentiation and death [11]. In non-stimulated conditions, ERK1/2 is anchored to the cell cytoplasm [51]. Nevertheless, mitogens promote activation and nuclear translocation of ERK1/2 for G1 to S phase progression of the cell cycle [51]. Melatonin modulates differently the ERK1/2 expression in tumor and non-tumor cells. In non-tumor cells, pineal hormone enhances the ERK1/2 expression and cell viability and inhibits apoptosis [52–54]. On cancer, nuclear ERK1/2 regulates several transcription factors leading to changes in gene expression and increased tumor growth [55]. A growing number of studies have shown that the ERK1/2 protein participates of several oncogenic mechanisms [56]. Pineal hormone acts by inhibiting ERK1/2 expression and the pro-tumorigenic effects of this molecule. On HNC, studies show that melatonin treatment inhibits cell proliferation, migration and invasion [3]. In addition, it may also induce mitochondria-dependent apoptosis and reduce tumor growth [14]. All these anti-tumorigenic effects are controlled by the ERK1/2-mediated signaling pathway suppression [3, 14]. Similar results have also been found in studies with lung [3, 57, 58], breast [59] cancers, leiomyosarcoma and osteosarcoma [60, 61].

In the current study, besides the increased ERK1/2 expression in tumors from PNT rats, we demonstrated that pinealectomy induced higher expression of nuclear p53 in the OSCC invasive front. Tumor suppressor p53 is an important transcription factor responsible for maintaining genomic stability [62]. In homeostatic conditions, p53 protein is maintained at low levels and the higher concentrations remains in the cell cytoplasm [63]. Following exposure to stress conditions, p53 protein regulates the activity of various signaling pathways involved with cell growth regulation, cell cycle progression, DNA repair and apoptosis [64]. TP53 gene mutations are the most common genetic alterations in the cancer [65]. These mutations may lead to synthesis and accumulation of dysfunctional proteins in the nucleus of tumor cells allowing the survival of genetically unstable cells [66]. Thus, although there is an increase in p53 expression, its function is impaired by the mutations. p53 nuclear protein overexpression is usually an indicative of the presence of mutant p53 and is commonly seen in OSCCs [67]. There are no studies that have evaluated the tumor p53 expression in tumors from pinealectomized rats. However, melatonin treatment can increase the activity of p53 tumor suppressor signaling pathway, reducing the tumor growth [33]. In the current study, we suggest that melatonin deficiency promoted by the pinealectomy induced an accumulation of dysfunctional p53 proteins in the nucleus of the tumor cells. In short, melatonin may increase the activity of p53 pathways and decrease the ERK1/2 activation. Although the cancer animals were not treated with melatonin, the results of this study suggest that the pinealectomy may promote chemically induced OSCC growth in rats through nuclear overexpression of ERK1/2 and dysfunctional p53.

Pineal hormone suppression may induce biobehavioral alterations in rodents [68, 69]. However, in our study, we hypothesized that pinealectomized rats would display increased depressive- and anxiety-like behaviors when compared to non-pinealectomized rats. Contrary to our expectations, there were no significant differences between the groups regarding the behavioral phenotype. In shift workers and people who stay up watching TV, using smartphones and/or suffering from insomnia, the artificial light exposure during the night period may inhibit melatonin production [70–72]. Furthermore, chronic tobacco smoking and alcoholism, the main risk factors for oral cancer development, are also associated with decreased systemic melatonin levels [73–75]. In the oral tissues, the melatonin produced by the pineal gland plays an important protective role against mechanisms associated to carcinogenesis, such as mucosa inflammation and oxidative damage [76, 77]. Melatonin treatment can inhibit the pro-tumorigenic action of lysine-specific demethylase 1 (LSD1), an enzyme that increases OSCC cell proliferation [57]. In oral cancer patients, we suggest that melatonin suppression would be correlated with a higher tumor progression, lower survival rate and worse response to oncological treatment. A clinical trial involving HNC patients showed that melatonin adjuvant treatment suppressed tumor growth and improved survival rate [4].

**Figure 6:**
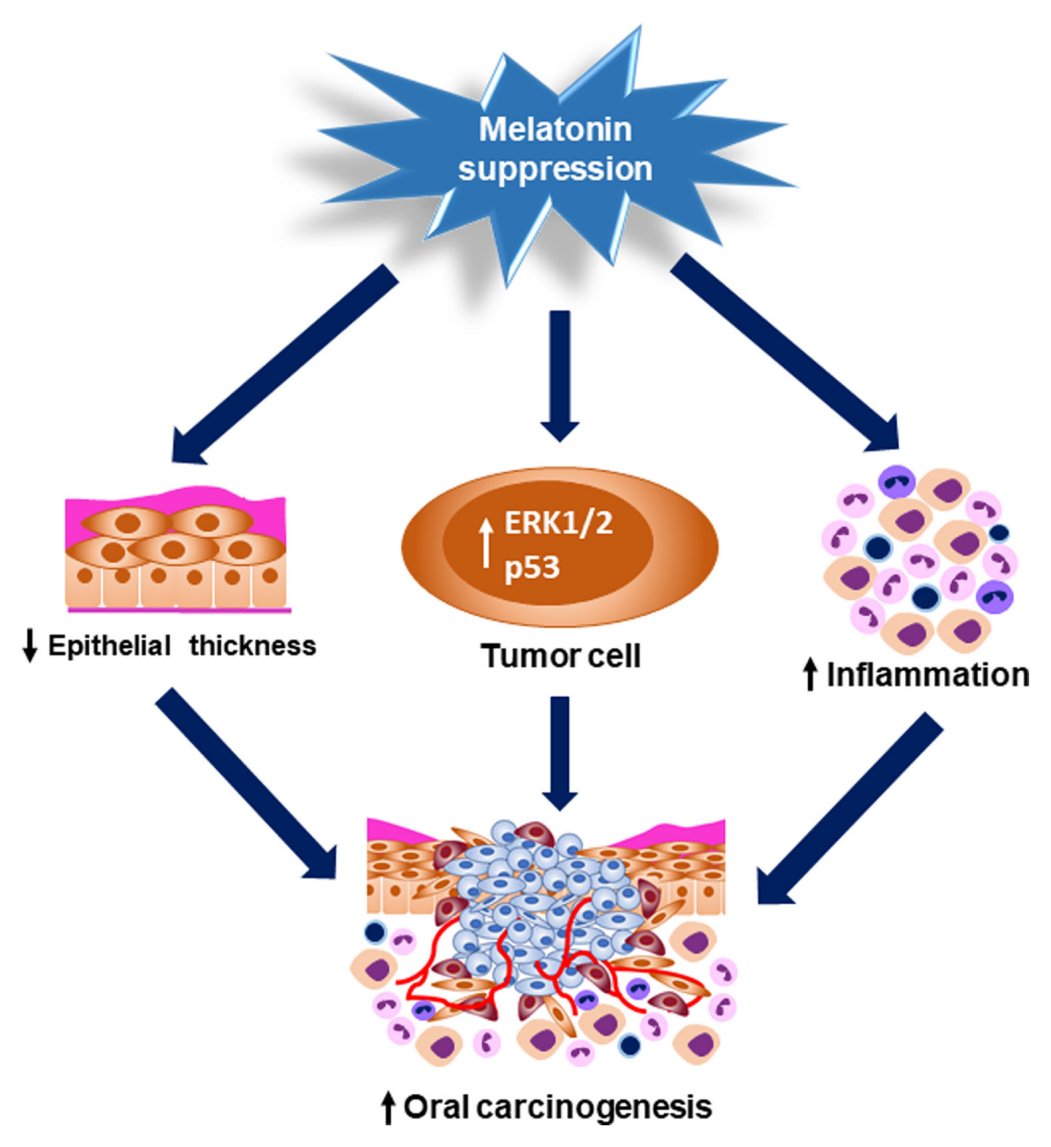
Graphical abstract showing the pinealectomy effects on OSCC occurrence and progression. Pinealectomy induced a significant reduction of the oral epithelium thickness, which may have increased the mucosa susceptibility to the chemical carcinogen. Moreover, melatonin suppression up-regulated the nuclear levels of ERK1/2 and p53 in the tumor cells and also increased the number of inflammatory cells in the tumor invasive front. All of these mechanisms could contribute to the oral carcinogenesis and tumor progression.

In humans, prospective studies that allow to evaluate the role of pineal hormone suppression on tumorigenesis require a large population sample with night habits and its long-term follow-up. Pinealectomy may be considered a useful method to evaluate the effects of melatonin suppression on cancer onset and progression in preclinical models due to the controlled laboratory conditions. Our research provides evidences about the protective role of pineal gland against chemically induced oral carcinogenesis and tumor progression. In view of our results, harmful habits that result in melatonin suppression could be considered as possible risk factors for HNC occurrence. One limitation of our study is the absence of a pinealectomized group and treated with melatonin in order to evaluate whether the pineal hormone could reverse the pinealectomy effects on cancer onset and progression. Moreover, the systemic melatonin levels were not measured in the groups of pinealectomized and sham-operated rats. The signaling pathways that modulate ERK1/2 and p53 proteins were also not evaluated in this study. Nevertheless, our research provides the first evidences on the role of pineal gland as biological protector against chemically induced oral carcinogenesis and tumor progression (Figure 6). In view of our results, harmful habits that result in decreased melatonin synthesis could be considered as possible risk factors for HNC occurrence.

## MATERIALS AND METHODS

### Animals and experimental design

In order to assess whether the pinealectomy and melatonin suppression affects oral cancer onset and progression, twenty-three male Wistar rats were housed in groups of 4 animals per cage (25.9 × 47.6 × 20.9 cm, polypropylene) and kept under standardized conditions (22 ± 2°C; 12/12 h light/dark cycle; lights on at 7:00 h). The animals were divided into 2 groups: PNT: 12 rats submitted to pinealectomy; Sham: 11 rats submitted to sham surgery. Fifteen days after pinealectomy or sham surgeries, all animals underwent chemically induced oral carcinogenesis for 16 weeks. The rats were also tested for depressive- and anxiety-like behaviors using FST and EZM, respectively. At the end of the experimental period, the animals were euthanized for evaluation of oral cancer occurrence and progression. All the experimental protocols were approved by the Animal Ethics Committee of the São Paulo State University, School of Dentistry, Araçatuba, SP, Brazil (Protocol Number: 00522-2017).

### Pinealectomy

Pinealectomy was performed as described by Hoffman & Reiter [78]. The rats were anesthetized with intraperitoneal injection of Ketamine Chlorhydrate 10% (50 mg/kg) and Xilazine Chlorhydrate 2% (5 mg/kg). A sagittal incision was performed on the scalp along the midline. Skin and muscles were pulled away to expose the lambdoid suture. A bone window was created with a circular trephine (5 mm in diameter) on the confluence of the superior sagittal and transverse venous sinuses. Then, the pineal gland was withdrawal and the bone-disk was replaced. The skin and muscles were sutured with surgical suture thread (Shalon^®^ 4-0). The animals of sham group were submitted to the same procedures; however, the pineal gland was not removed. Pineal glands from PNT group rats were fixed in 10% buffered formaldehyde solution (Merck, Darmstadt, Germany) for 48 h. Then, they were dehydrated and paraffin embedded. The histological sections were obtained and stained with hematoxylin and eosin (H&E). Morphological analysis of tissue was performed by a pathologist to prove that it was in fact the pineal gland.

### Behavioral phenotyping

Behavioral evaluation was accomplished in two phases of the experimental period: 1) two weeks after the surgery (before starting carcinogenic induction) and 2) after carcinogen treatment. Depressive- and anxiety-like behaviors of each experimental group were evaluated by a blinded experienced observer. *Forced swimming test*: FST was accomplished as described by Porsolt et al. [79]. Firstly, the rats were forced to swim for 15 minutes in a glass cylinder (13 cm in diameter × 24 cm in height) filled with water (25ºC ± 2) (pre-test). On the next day, the rats were forced to swim for 6 minutes at the same pre-test conditions. FST was recorded with a camera positioned 100 centimeters away from the glass cylinder. To evaluate the depressive-like behavior, immobility time of each animal was analyzed. The immobility time was considered as absence of escape-oriented behaviors [30]. *Elevated zero maze*: EZM is an annular platform elevated 50 cm above the floor. The apparatus has a diameter of 105 cm divided into two opposite open arms and two opposite closed arms. The rats were placed individually into one of close arms and their behaviors were recorded for 10 minutes with a camera positioned above of apparatus. The anxiety-like behavior of each animal was analyzed by the time spent in the open arms [80].

### Oral carcinogenesis model

Two weeks after the pinealectomy or sham surgery, all rats were submitted to oral carcinogenesis. For tumor induction, the rats were treated with 50 ppm of 4-nitroquinoline-1-oxide (4NQO) (Sigma-Aldrich, St. Louis, MO, USA) diluted in drinking water [81]. All animals had free access to food (Purina^®^, Paulínia-SP, Brazil) and carcinogen solution, which were replenished twice a week. After 16 weeks of carcinogen treatment, the rats were euthanized and their tongues with carcinogen-induced lesions were removed to perform histopathological and molecular analyzes as well immunohistochemistry reactions.

### Histopathological analysis

The tongues were longitudinally sectioned and fixed in 10% buffered formaldehyde solution (Merck, Darmstadt, Germany) for 48 h. The tissues were alcohol dehydrated and paraffin embedded. Histological sections (3 μm of thickness) were obtained and stained with hematoxylin and eosin (H&E). The tongue lesions were classified in oral leukoplakia or OSCC [82]. OSCC was classified as well-, moderately- or poorly differentiated, according to the WHO classification [83]. Leukoplakia is a precursor lesion of OSCC. The following microscopic features were considered for the diagnosis of oral leukoplakia: epithelial atrophy, acanthosis with or without hyperkeratosis and epithelial dysplasia [84]. The degree of epithelial dysplasia was classified into mild, moderate or severe [83]. Microscopic examination was performed by an experienced oral pathologist who was blinded to the experimental groups.

### Tumor thickness and volume

The three-dimensional measurements of the tumor were obtained with a digital caliper. The tumor volume was calculated in mm^3^ using the formula: depth × width × length [82]. To evaluate the tumor thickness, OSCC slides were photographed on a microscope equipped with a digital camera (Zeiss Axio imager Z1 microscope, Carl Zeiss, Munchen-Hallbergmoos, Germany). The measurements were obtained in μm from the deepest point of tumor invasion to the surface of the lesion using the ImageJ software [82].

### Non-tumor epithelial thickness

The non-tumor epithelium thickness adjacent to the tongue lesions was obtained in μm by the distance between basal cell layer and granular layer. Corneal thickness was assessed by the measure between the lucid layer and corneal layer surface. Total epithelial thickness was measured between the basal cell layer and apical epithelial surface (epithelium + corneal layer) [85]. All measurements were performed by a blinded researcher to the experimental groups. On each slide, 4 fields were analyzed: two fields immediately adjacent to the tongue lesions and two distant fields from the lesion site. For statistical analysis, the mean thickness of epithelium and corneal layer were calculated between two fields immediately adjacent to the tongue lesions and between two distant fields from the lesion.

### Inflammatory cells quantification in the tumor invasion front

To evaluate the influence of melatonin suppression on tumor inflammatory response in sham and PNT rats, the average number of neutrophils, eosinophils, macrophages, lymphocytes and mast cells were quantified in OSCC slides stained with H&E, at 1000× magnification (Leica DM2500, Leica Biosystems, Wetzlar, Germany). On each slide, five fields of tumor invasion front were assessed. The analysis was performed by a blind researcher to experimental groups.

### Immunohistochemistry

Histological sections of OSCC were deparaffinized and rehydrated to immunohistochemistry evaluation. Heat-induced epitope retrieval was performed by 10 mM citrate buffer, pH 6.0, at 55°C for 20 minutes. Blockade of endogenous peroxidase activity was accomplished by 3% H_2_O_2_ for 20 minutes. The slides were washed with PBS solution (pH 7.2). Sections were then incubed with primary antibody anti-p53 (dilution 1:100; Santa Cruz Biotechnology, Santa Cruz, CA, USA), anti-PKA (dilution 1:3000; Santa Cruz Biotechnology, Santa Cruz, CA, USA) and anti-ERK1/2 (dilution 1:200; Santa Cruz Biotechnology, Santa Cruz, CA, USA) at 4°C overnight. The sections were incubated with Histofine antibody polymer conjugated with horseradish peroxidase (Nichirei Biosciences, Tokyo, Japan) for 30 minutes. The slides were washed with PBS buffer and a chromogenic substrate (3,3′,5,5′-tetramethylbenzidine) was incubated for 5 minutes. Reactions were stopped in deionized water. Counter-staining was performed by Harris’s hematoxylin for 20 seconds. The histological sections were dehydrated and covered with coverslip for microscopic examination. In the tumor invasive front, a total of 1000 tumor cells was assessed in 2–4 fields at 400× magnification. The percentage of immunopositive cells was used to express the results. All the analyzes were performed by a blinded examiner to the experimental conditions.

### Expression of tumor progression-related genes and melatonin receptors

Real time-PCR technique was performed to assess the mRNA expression levels for VEGF, NFkB, MMP-2, MMP-9 and CDKN2a-p16 in the tumor microenvironment. These genes were chosen because they are associated to oral cancer development and progression. VEGF promotes tumor angiogenesis [86]. MMP-2 and -9 are known to influence degradation of the extracellular matrix and contribute to tumor invasion and metastasis [87, 88]. CDKN2a-p16 is an important tumor-suppressor gene often associated to oral cancer development [89]. NFkB is a proinflammatory transcription factor that plays a pivotal role in tumorigenesis and oral cancer progression [90]. The mRNA expression levels for MTNR1a and MTNR2a melatonin receptors were also examined in the tumors from both experimental groups. The tumor specimens were washed in saline solution, immersed in TRIzol (Invitrogen Life Technologies, USA) and stored at –80ºC. Total RNA was extracted for synthesis of complementary DNA (cDNA). RNA quantity and quality were evaluated by Nanodrop 2000 (Thermo Scientific, Wilmington, USA). cDNA was synthesized using the High Capacity RNA to cDNA kit (Invitrogen Life Technologies). TaqMan™ RT-PCR assay measured the mRNA expression levels by the StepOne Real-Time PCR system (Applied Biosystems, Foster City, CA, USA). The primers used were VEGF (Rn01511601_m1), NFkB (Rn01310378_g1), MMP–2 (Rn01538170_m1), MMP–9 (Rn00579162_m1), CDKN2a-p16 (Rn00580664_m1), MTNR1a (Rn01488022_m1) and MTNR1b (Rn01447987_m1). β-actin (Rn00562253_m1) gene was used as endogenous control. mRNA Relative Quantity (RQ) for each target gene was calculated using the comparative Ct method. The assays were performed in duplicate.

### Statistical analysis

GraphPad Prism 6.01 software (GraphPad Software Inc., San Diego, CA, USA) was used to perform the statistical analysis. Chi-square test was performed to assess the OSCC incidence in both experimental groups. Student’s *t*-test was used to determine the differences between PNT and sham-operated rats regarding the tumor volume and thickness, epithelial thickness, number of inflammatory cells, mRNA and protein levels and behavioral measures. Analysis of variance (One-way ANOVA) with post-test Tukey–Kramer multiple comparison analysis was performed to determine the differences between the mean of inflammatory cells in the tumor invasion front of sham animals and early- or advanced tumor-bearing PNT rats. The level of statistical significance was set at a *p* value less than 0.05 (*p* < 0.05) for all statistical tests.

## CONCLUSIONS

Taken together, our results reveal for the first time that pinealectomy followed by melatonin suppression may induce higher oral cancer occurrence and progression in a preclinical model. Our findings suggest that chemically induced OSCC development can be influenced by decreased epithelial thickness after pinealectomy. Furthermore, the accelerated tumor progression in pinealectomized rats could be mediated by increase of tumor-associated macrophages and eosinophils and ERK1/2 oncogenic protein, besides p53 inactivation.

## Abbreviations

OSCC: Oral squamous cell carcinoma
ERK1/2: Extracellular signal-regulated kinases
HNC: Head and Neck Carcinoma
MMP-2: Matrix Metalloproteinase-2
MMP-9: Matrix Metalloproteinase-2
NF-kB: Factor Nuclear kappa B
VEGF: Vascular endothelial growth fator
DMBA: 7,12-Dimethylbenz(a)anthracene
PNT: Pinealectomized
WHO: World Health Organization
FST: Forced swimming test
EZM: Elevated Zero Maze
CP4501B1: Cytochrome P 4501B1
NAS: N-acetylserotonin
acetyl-CoA: Acetyl coenzyme A
ROS: Reactive oxygen species
PCNA: Proliferating cell nuclear antigen
HIF-1α: Hypoxia inducible factor-1α
4NQO: 4-Nitroquinoline 1-oxide
mRNA: Messenger RNA
MT1: Melatonin receptor 1
MT2: Melatonina receptor 2
PKA: Protein Kinase A
MAPK: Mitogen-activated protein kinase
DNA: Deoxyribonucleic acid
LSD1: lysine-specific demethylase 1
ppm: Parts per million
μm: Micrometer
H&E: Hematoxylin and Eosin
mm^3^: Cubic Millimeters
H_2_O_2_: Hydrogen Peroxide
PBS: Phosphate buffer Solution
PCR: Polymerase Chain Reaction
RNA: Ribonucleic acid
cDNA: Complementary DNA
RT-PCR: Real Time Polymerase Chain Reaction
